# Association between Outpatient Visits and Initiating Medication among Elderly Patients after an Osteoporotic Vertebral Fracture

**DOI:** 10.3390/jcm11175035

**Published:** 2022-08-27

**Authors:** Ya-Chi Wu, Chuan-Yu Chen, Chin-Sung Chang, Chia-Chun Li, Ru-Hsueh Wang, Zih-Jie Sun, Chih-Hsing Wu, Yin-Fan Chang

**Affiliations:** 1 Department of Family Medicine, An Nan Hospital, China Medical University, Tainan 70965, Taiwan; 2 Department of Family Medicine, National Cheng Kung University Hospital, College of Medicine, National Cheng Kung University, Tainan 70403, Taiwan; 3 Institute of Allied Health Sciences, College of Medicine, National Cheng Kung University, Tainan 70101, Taiwan; 4 Division of Family Medicine, National Cheng Kung University Hospital Dou Liu Branch, Yunlin 64043, Taiwan; 5 Department of Family, College of Medicine, National Cheng Kung University, Tainan 70101, Taiwan; 6 Institute of Gerontology, College of Medicine, National Cheng Kung University, Tainan 70101, Taiwan

**Keywords:** vertebral fracture, treatment gap, initiating treatment, outpatient visit

## Abstract

Purpose: A treatment gap exists in vertebral fracture (VF) patients. An outpatient visit is a necessary step to initiate treatment. The study aimed to evaluate factors associated with an outpatient visit following a VF diagnosis, and the association between the interval of an outpatient visit after VF diagnosis and its impact on prescribing of anti-osteoporosis medications (AOMs). Methods: Subjects 65 years and older from Tianliao Township in Taiwan with newly diagnosed VF between 2009 and 2010 were included. Information about outpatient visits and AOMs prescriptions were derived from the National Health Insurance Research database and followed up for 2 years. Factors associated with outpatient visits and the initiation of AOMs were assessed using the multivariable Cox proportional regression model analysis. The receiver operating characteristic curve (ROC curve) was analyzed to determine the predictive effects of the interval between an outpatient visit following the diagnosis of a new VF on initiating AOMs and the potential optimal cutoff point. Results: Of 393 participants, 42.2% had outpatient visits within 2 years after a new VF diagnosis, for which the mean interval was 4.8 ± 4.8 months. Patients who were female and reported a current use of supplements were positively associated with visits after a new VF diagnosis, but the bone mineral density (BMD) T-score was negatively associated with visits. Furthermore, 140 (35.6%) patients had initiated AOMs within 2 years after the diagnosis of a new VF. It was found that a higher BMD T-score and a longer interval between an outpatient visit following diagnosis was negatively associated with initiation of AOMs. The ROC curve analysis showed outpatient visits within 3 months after a VF diagnosis had the highest Youden index and maximum area under the curve. Conclusions: Patients who were female, were currently taking supplements, and those who had a lower BMD T-score were more likely to visit doctors after being diagnosed with a new VF. Furthermore, a lower BMD T-score and a shorter interval, within 3 months and not more than 8 months, between an outpatient visit following the diagnosis of VF increased the likelihood of being prescribed AOMs.

## 1. Introduction

Osteoporosis is a progressive systemic skeletal disease related to reduced bone mass that leads to an increased risk of fractures [1], especially at the region of hip, vertebra, wrist and distal forearm [2]. It is so known as a “silent” disease due to a lack of symptoms. Therefore, osteoporosis has always been underestimated and underdiagnosed, especially in the case of vertebral fractures. Two-thirds of vertebral fractures are under-diagnosed due to a lack of apparent symptom in medical practice [3]. Thus, under-diagnosis of vertebral fractures becomes a worldwide problem.

The ratio of under-diagnosed vertebral fractures assessed by a thoracolumbar lateral radiograph varies in different area, for example, 46% in Latin America, 45% in North America, and 29% in Europe/South Africa/Australia [4]. In Taiwan, the prevalence rate of vertebral fractures in women older than 65 is 20%, and that for men is 12.5% [5]. In addition, vertebral fractures increase the risk of following fractures, morbidities, and mortality. 20% of osteoporotic postmenopausal women experience a following vertebral fracture within one year after an initial vertebral fracture and also suffer from high risk of increases in other major osteoporotic fractures, such as hip fractures [6]. After a painful vertebral fracture, the need for primary care services is 14 times greater in postmenopausal women as compared to the general public in the first year following a fracture. Furthermore, vertebral fractures have been related to 15% higher mortality rate [6].

Fortunately, anti-osteoporosis medications (AOMs) play a big role in preventing secondary fragility fractures [7]. However, most patients who suffer from a fragility fracture don’t acquire sufficient treatment [8,9] because a treatment gap occurred as a result of under-diagnosis and under-treatment [10]. Initiating osteoporosis treatment is a complex process that includes interactions among patients, doctors, and patient awareness. The factors associated with initiating AOMs have been discussed in previous studies [11,12,13,14,15,16] and include sex, age, race, BMI, underlying diseases, smoking, previous fracture or fall experience, bone density, supplement intake, and socioeconomic status. However, some previous studies have included subjects from hospitals, especially from emergency rooms. In one study, data from a single hospital only included one-third of subjects with vertebral fractures and thus could not provide an entire view of the treatment gaps. Furthermore, most previous studies have used big data, such as health insurance data, where diagnosis of osteoporosis and vertebral fractures were defined using ICD numbers, and detailed factors associated with treatment were limited [11,12,13,14,15,16].

Due to the lack of obvious symptoms of vertebral fractures, we used data from a Tianliao community study to evaluate factors associated with outpatient visits after vertebral fractures. We also focused on the association between the interval of an outpatient visit after vertebral fracture diagnosis and whether AOMs were prescribed.

## 2. Methods

### 2.1. Participants

**Figure 1:**
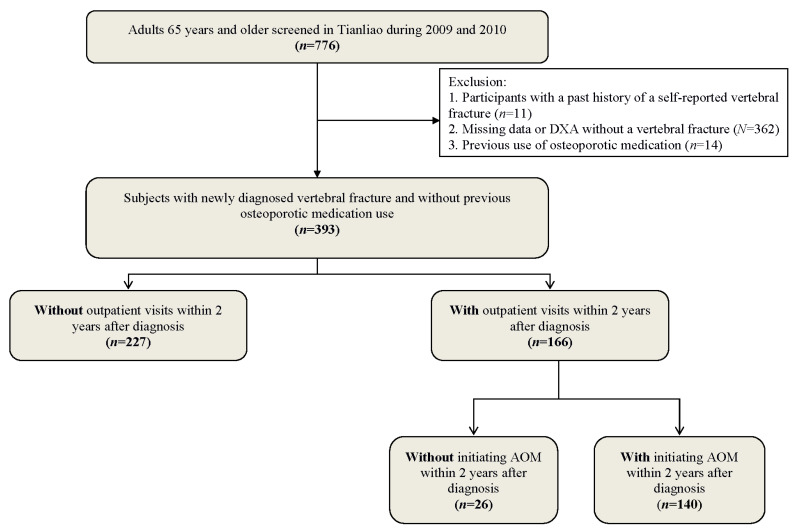
Flow chart of study design, *n* = 393.

In total, 776 subjects aged 65 years and above were randomly selected in Tianliao Township between 2009 and 2010 [17,18]. After excluding self-reported vertebral fracture history (*n* = 11), use of anti-osteoporotic medications (*n* = 14), missing data (*n* = 28), and diagnosis without vertebral fracture by thoracolumbar spine lateral view X-ray (*n* = 334), a total of 393 subjects with newly X-ray diagnosed vertebral fractures were enrolled in the final analysis (Figure 1). This study was confirmed by the Institutional Review Board (IRB) of National Cheng Kung University Hospital (IRB number: A-ER-106-123), and informed consent was achieved consequently.

A well-trained research assistant helped each subject to complete the structured questionnaires by interview [17] and physical examination, including body mass index (BMI) and bone mineral density (BMD) values. The questionnaires involved several fields: sociodemographic characteristics (age, gender, and education level), habitual behavior (alcohol consumption, cigarette smoking, vitamin, calcium and glucosamine supplement use), medical history (thyroid disease, osteoarthritis, diabetes mellitus, rheumatoid arthritis), the Charlson comorbidity index (CCI) score [19,20], and osteoporosis-associated risk factors (family history of hip fractures, history of fracture, and use of steroids). The clinical risk factors for osteoporosis were evaluated on the basis of a fracture risk assessment tool (FRAX) [18].

Current smoking was defined as someone who had smoked more than 100 cigarettes and was still smoking [21]. Current drinking was defined someone as who had drunk 3 or more units of alcohol in one day for more than 6 months before the study [18,22]. A unit of alcohol was defined as equivalent to 10 g of alcohol. This is equal to a standard glass of beer (285 mL), a single measure of spirits (30 mL), 1 measure of an aperitif (60 mL), or a medium-sized glass of wine (120 mL). Supplement intake was defined as currently using vitamin, calcium, or glucosamine supplements, regardless of the duration or amount.

### 2.2. Data Collection

Taiwan launched a single payer national health insurance (NHI) program on 1 March 1995. Almost 99% of the public was registered in this program by 2007 [23]. The National Health Research Institutes manage Taiwan’s National Health Insurance Research Database (NHIRD) which can be used for research purposes. NHIRD is one of the largest nationally population-based databases all over the world. It involved important information, including the characteristics of the patients, hospitals, and physicians, as well as all clinical services received by each enrollee [24].

Data for outpatient visits and anti-osteoporotic medication prescriptions were obtained from NHIRD provided by the Health and Welfare Data Science Center and were followed up for 2 years after the Tianliao screening from 2009–2012. We assessed outpatient visits for osteoporosis or vertebral fracture by identifying the International Classification of Diseases (ICD) code algorithms (osteoporosis: ICD-9: 733.0–733.1, ICD-10: M80, M81, and vertebral fracture: ICD-9: 805.2–805.9, 733.13, ICD-10: S22.0–S22.1, S32.0–S32.2). AOMs were defined as receiving osteoporosis medication, including alendronate, risedronate, ibandronate acid, zoledronic acid, denosumab, raloxifen, bazedoxifen, calcitonin, and teriparatide.

### 2.3. BMD Measurement

The BMDs were assessed using dual-energy X-ray absorptiometry (DXA) (QDR Explorer; Hologic, Sunnyvale, CA, USA) settled in a mobile bus, including the lumbar spine (L1–L4) and hip (total hip and neck) regions. The precision error derived from repeated lumbar, total hip, and neck BMD measurements taken from 30 female subjects were 1.15%, 1.42%, and 1.51%, respectively [17]. The least significant changes were 3.19%, 3.93%, and 4.18% for each region, respectively. The scanning procedures and precision were all within the requirements of the International Society for Clinical Densitometry official position statement [25].

### 2.4. Vertebral Fracture Assessment

A single radiologist interpreted the thoracolumbar spine lateral view X-rays (T4 to L6) blindly. To classify vertebral fractures, a combination of the Genant et al. semiquantitative (SQ) method and morphometry were used: grade 1 = a reduction in vertebral height of 20–25%, grade 2 = a reduction of 26–40%, and grade 3 = a reduction of more than 40% [26].

### 2.5. Statistical Analysis

All statistical analyses were achieved using SAS^®^ software, version 9.4 (SAS Institute Inc., Cary, NC, USA). A *t*-test was performed to ascertain the mean differences and continuous variables were expressed as mean ± standard deviation. Categorical variables were stated as proportionate percentiles, and a chi-square test was used to determine the proportional differences. A multivariable Cox proportional regression model analysis was applied to identify the independence of factors associated with outpatient visits and initiating AOMs. In addition, an attempt was made to assess the predictive effects of the interval between an outpatient visit following diagnosis on whether initiating AOMs and the potential optimal cutoff point by analyzing the ROC curve. Significance was set at *p* < 0.05 (two-tailed).

## 3. Results

Of 393 participants, 45% were female, and the mean age was 75.1 ± 6.0 years. The mean BMI was 24.5 ± 3.4 kg/m^2^, and the mean BMD T-score was −2.0 ± 1.2. Furthermore, 42.2% had outpatient visits within 2 years after a diagnosis of a new vertebral fracture, and the mean interval was 4.8 ± 4.8 months. Subjects who were female (75.9% vs. 21.6%), older (mean age: 76.0 ± 5.6 vs. 74.4 ± 6.2), not current smoker (3.6% vs. 14.1%), current use of supplements (45.8% vs. 27.8%), had history of more previous fractures (27.1% vs. 16.0%), and had lower BMD T-score (−2.74 ± 1.01 vs. −1.46 ± 1.02) were more likely to visit a doctor within 2 years after being diagnosed with a new vertebral fracture (Table 1).

Using a multivariable Cox proportional regression model adjusted for gender, age, BMI, previous history of fractures, parent history of hip fractures, current smokers, current alcohol consumption, a history of steroid and supplement use, a higher Charlson comorbidity index and BMD T-score, it was found that being female (OR = 6.66, 95% CI: 3.52–12.60, *p* < 0.001) and current use of supplements (OR = 2.14, 95% CI: 1.22–3.74, *p* < 0.005) were positively associated with visits after a newly diagnosed vertebral fracture, but the BMD T-score was negatively associated with visits (OR = 0.43, 95% CI: 0.31–0.59, *p* < 0.001) (Table 2).

Of 393 participants, 140 (35.6%) initiated anti-osteoporotic medications within 2 years after a diagnosis of a new vertebral fracture. Subjects who were female (80.7% vs. 24.5%), older (76.2 ± 5.7 vs. 74.5 ± 6.1), current use of supplements (46.4% vs. 29.0%), not current smoker (2.1% vs. 13.8%), had lower T-score of BMD (−2.93 ± 0.85 vs. −1.49 ± 1.04), and had shorter interval between an outpatient visit following the initial diagnosis (4.0 ± 3.4 vs. 9.5 ± 8.0) were more likely to initiate anti-osteoporotic medication (Table 3).

Using a multivariable Cox proportional regression model adjusted for gender, age, BMI, previous history of fracture, current smoking, a history of supplement use, a Charlson comorbidity index, BMD T-score, and interval between an outpatient visit following the initial diagnosis of a new vertebral fracture, it was found that a higher BMD T-score (OR = 0.33, 95% CI: 0.16–0.68, *p* < 0.005) and a longer interval between an outpatient visit following diagnosis (OR = 0.86, 95% CI: 0.78–0.96, *p* < 0.05) was negatively associated with initiation of anti-osteoporotic medications (Table 4).

**Figure 2:**
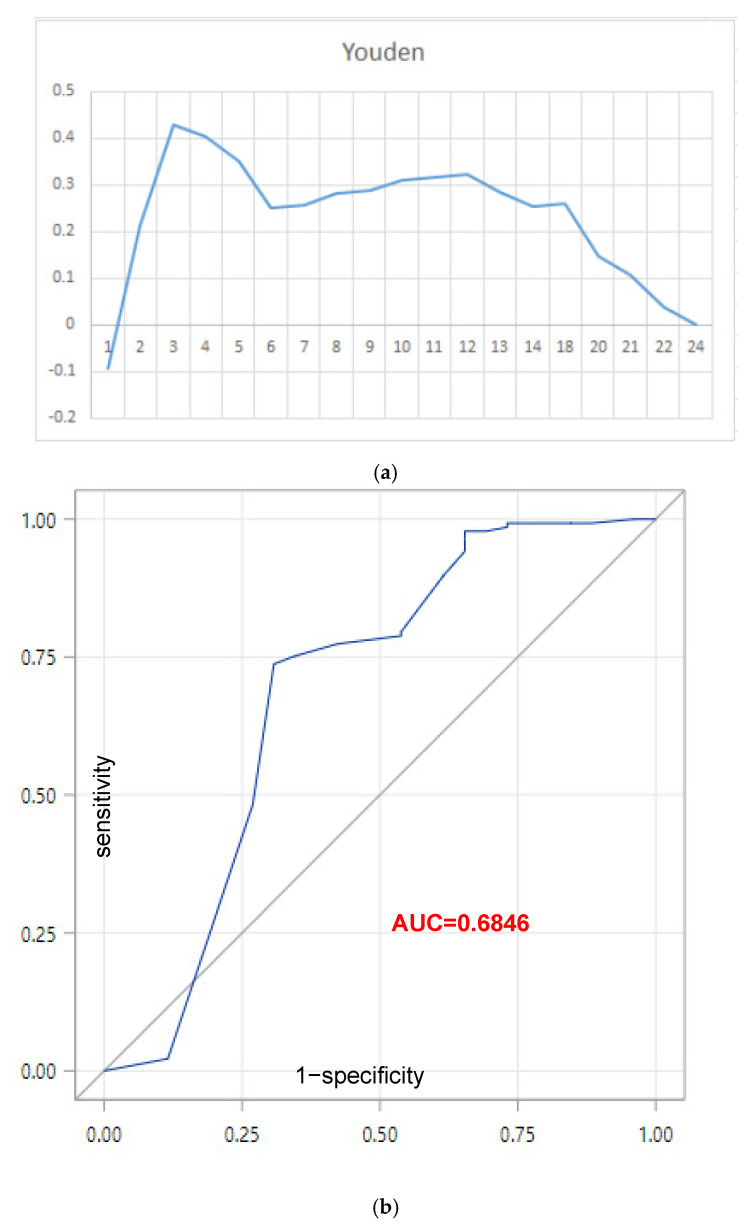
In (**a**,**b**), using receiver operating characteristic (ROC) curve analysis used to evaluate the association between initiating anti-osteoporotic medication and the interval between an outpatient visit following a new diagnosis of a vertebral fracture. The figure shows that outpatient visits within 3 months had the highest Youden index (0.43) and the highest maximum area under the curve (AUC = 0.6846). (**a**) Youden index, (**b**) When the cutoff point was 3, the maximum AUC was 0.6846.

We used a receiver operating characteristic (ROC) curve analysis to find appropriate cutoff values between initiation of anti-osteoporotic medication and the interval between an outpatient visit following the initial diagnosis of a new vertebral fracture. It was found that outpatient visits within 3 months after a vertebral fracture diagnosis had the highest Youden index (0.43) and maximum area under the curve (AUC = 0.6846) (Figure 2). Furthermore, we also found that an outpatient visit within 8 months following diagnosis increased the likelihood of initiating AOMs (OR 7.44, 95% CI 2.54–21.81) but visits within 9 months did not (OR 2.82, 95% CI 0.82–9.70, data not shown). Therefore, the interval for outpatient visits should be within 3 months and no more than 8 months after diagnosis of a new vertebral fracture to increase the likelihood of initiating AOMs.

## 4. Discussion

Initiating treatment is a necessary first step in the management of osteoporosis. In our study, the treatment rate within 2 years for patients after newly diagnosed vertebral fracture was 35.6%. In an Indian study, the treatment rate within 2 years for subjects aged 50 years old and above post osteoporotic fracture was 23.3% [13]. The fracture risk Brussels epidemiological enquiry revealed that the treatment rate within 1 years for women aged 60 years old and above post vertebral fracture was 29.5% [27]. The international costs and utilities related to osteoporotic fractures study (ICUROS) in North America, Europe, and Australia showed that the treatment rate within 1.5 years for subjects aged 50 years old and above post vertebral fracture was 33.0% [28]. The treatment rate varies due to different age group, fracture type, and observed period. Our study revealed similar treatment rate. Therefore, low treatment rate is a crucial public health issue, not only in Taiwan, but also in worldwide.

The biggest difference between our study and previous studies was that we divided the process of initiate AOMs into 2 steps. We supposed that initiate AOMs should be happened after an outpatient visit as diagnosis of newly vertebral fracture. We discussed associated factors of outpatient visit after diagnosis of newly vertebral fracture at first, then associated factors of initiate AOMs was analyzed. Most of previous studies mixed 2 steps together. To the best of our knowledge, just one study presented outpatient visit after diagnosis of fracture [29], but detailed associated factors were not discussed. Several studies showed that factors of female, older age [15], higher daily calcium intake [11,14], history of fall [16], higher income [14] and lower T-score [30] have higher rate of initiating AOMs. On the contrary, men, black race [13], body mass index ≥ 30, current tobacco use, history of arthritis [14], and higher Charlson comorbidity index scores [12] decreased the rate of initiating AOMs. However, our study showed that female, current supplement use and lower T-score of BMD were associated factors of higher rate with outpatient visit, not so-called associated factors of initiating AOMs. The “true” associated factor of initiating AOMs were T-score of BMD and interval between an outpatient visit following the initial diagnosis.

The International Osteoporosis Foundation (IOF) has developed a fracture liaison service (FLS) for the treatment of osteoporotic patients. The primary objectives of the FLS are to establish critical procedures that ensure identification, assessments, initiation of treatment, and tracking of fracture patients. Thirteen criteria are described in the best practice framework standard documents to guide healthcare systems and assist them with focusing on key priorities [31].

It is suggested that post fracture assessment timing, such as BMD testing, should be within 8 weeks and no more than 16 weeks after a fracture diagnosis. The timing of post fracture assessment is similar to the findings of the present study. We suggest that an outpatient visit should be scheduled within 3 months and no more than 8 months after a vertebral fracture.

Our study has several limitations: First, recall bias may occur [17]. The subjects completed the questionnaires by self-reporting of data. Recall bias exists when something occurred 20 years prior or with more complicated behavior. However, recall bias may be decreased if memories were simply engraved, such as disease history and long-term lifestyle [32], but detailed information about the amount of supplement use couldn’t be obtained in our study. Second, some of anti-osteoporotic medications and the supplements, such as vitamin, calcium, or glucosamine, were self-paid. Neither of them were recorded in the NHIRD. Accordingly, we could not trace the use of supplements and self-paid medications during the period of the study. To put it another way, patient might in fact have obtained AOMs out of pocket but recorded as not receiving AOM treatment in the NHIRD. Therefore, the estimation of subjects taking AOMs may have been underestimated. Moreover, we didn’t collect the blood data about vitamin D, calcium or PTH, which might affect therapeutic effectiveness and probability of re-fractures. However, the outcomes of our study were with/without the outpatient visits and anti-osteoporotic medication prescriptions. We hold that use of supplements during the period of the study did not influence our outcomes. Finally, our study concerned with adults aged above 65 in a rural community with a high prevalence of osteoporosis. Moreover, this process could differ in countries/populations/ethnic groups due to variety of expectancies. Further research is required to provide evidence for general population.

In conclusion, we found that female subjects, current use of supplements, and lower BMD T-scores made it more likely that patients would visit a doctor after being diagnosed with a new vertebral fracture. Furthermore, a lower BMD T-score and a shorter interval between outpatient visits following diagnosis increased the likelihood of initiating AOMs. Clinical services providers should encourage patients to follow up at outpatient departments within 3 months and not more than 8 months after a vertebral fracture.

## Figures and Tables

**Table 1:** Demographic characteristics of 393 subjects with osteoporotic vertebral fractures.

	Outpatient Visits within 2 Years after Diagnosis	
	without *n* = 227	with *n* = 166
Gender, female ***	49 (21.6) ^#^	126 (75.9)
Age, years *	74.4 ± 6.3	76.0 ± 5.6
Body mass index, kg/m^2^	24.7 ± 3.4	24.3 ± 3.4
Previous fracture **	36 (16.0)	45 (27.1)
Parent with fractured hip	9 (4.0)	9 (5.4)
Current smoking ***	32 (14.1)	6 (3.6)
Current alcohol consumption	10 (4.4)	6 (3.6)
Current steroid use	14 (6.2)	14 (8.4)
Current supplement use ***	63 (27.8)	76 (45.8)
Charlson comorbidity index score	1.46 ± 1.85	1.39 ± 1.59
T-score for bone mineral density ***	−1.46 ± 1.02	−2.74 ± 1.01
Interval between outpatient visits following diagnosis in months	-	4.8 ± 4.8

^#^: number (percentage) in categorical variables, χ^2^ test; mean ± SD in continuous variables, Student’s *t* test; * *p* < 0.05; ** *p* < 0.005; *** *p* < 0.001.

**Table 2:** Multivariable Cox proportional regression of the associated factors for outpatient visits.

	Outpatient Visits within 2 Years after Diagnosis
	OR (95% CI)
Gender, female vs. male	6.66 (3.52–12.60) ***
Age, years	1.03 (0.99–1.08)
Body mass index, kg/m^2^	0.97 (0.89–1.05)
Previous fracture	1.80 (0.93–3.46)
Parent with fractured hip	0.99 (0.28–3.42)
Current smoking	1.14 (0.40–3.25)
Current alcohol consumption	2.45 (0.69–8.66)
Current steroid use	0.87 (0.32–2.35)
Current supplement use	2.14 (1.22–3.74) **
Charlson comorbidity index score	1.09 (0.93–1.27)
T-score for bone mineral density	0.43 (0.31–0.59) ***

OR (95% CI): odds ratio (95% confidence interval); ** *p* < 0.005; *** *p* < 0.001.

**Table 3:** Demographic characteristics of 393 subjects with or without initiating anti-osteoporotic medication within 2 years after diagnosis.

	Initiating Anti-Osteoporotic Medication	
	without *n* = 253	with *n* = 140
Gender, female ***	62 (24.5) ^#^	113 (80.7)
Age, years *	74.5 ± 6.1	76.2 ± 5.7
Body mass index, kg/m^2^	24.7 ± 3.5	24.3 ± 3.3
Previous fracture	45 (17.9)	36 (25.7)
Parent with fractured hip	13 (5.2)	5 (3.6)
Current smoking ***	35 (13.8)	3 (2.1)
Current alcohol consumption	11 (4.4)	5 (3.6)
Current steroid use	15 (6.0)	13 (9.3)
Current supplement use ***	74 (29.0)	65 (46.4)
Charlson comorbidity index score	1.51 ± 1.85	1.29 ± 1.52
T-score for bone mineral density ***	−1.49 ± 1.04	−2.93 ± 0.85
Interval between an outpatient visit following diagnosis, months **	9.5 ± 8.0	4.0 ± 3.4

^#^: number (percentage) in categorical variables, χ^2^ test; mean ± SD in continuous variables, Student’s *t* test; * *p* < 0.05; ** *p* < 0.005; *** *p* < 0.001.

**Table 4:** Multivariable Cox proportional regression of the factors associated with initiating anti-osteoporotic medication (*n* = 393).

	Initiating Anti-Osteoporotic Medication
	OR (95% CI)
Gender, female vs. male	0.67 (0.18–2.44)
Age, years	1.08 (0.96–1.20)
Body mass index, kg/m^2^	1.03 (0.86–1.22)
Previous fracture	0.65 (0.20–2.09)
Current smoking	0.53 (0.06–4.37)
Current supplement use	1.42 (0.47–4.30)
Charlson comorbidity index score	0.79 (0.59–1.06)
T-score of bone mineral density	0.33 (0.16–0.68) **
Interval between an outpatient visit following diagnosis, months	0.86 (0.78–0.96) *

OR (95% CI): odds ratio (95% confidence interval), * *p* < 0.05; ** *p* < 0.005.

## Data Availability

Restrictions apply to the availability of these data. Data was obtained from the Health and Welfare Data Science Center and were available from Chih-Hsing Wu with the permission of the Health and Welfare Data Science Center.
